# Development of an Icariin-Loaded Bilosome-Melittin Formulation with Improved Anticancer Activity against Cancerous Pancreatic Cells

**DOI:** 10.3390/ph14121309

**Published:** 2021-12-15

**Authors:** Nabil A. Alhakamy, Shaimaa M. Badr-Eldin, Waleed S. Alharbi, Mohamed A. Alfaleh, Omar D. Al-hejaili, Hibah M. Aldawsari, Basma G. Eid, Rana Bakhaidar, Filippo Drago, Filippo Caraci, Giuseppe Caruso

**Affiliations:** 1 Department of Pharmaceutics, Faculty of Pharmacy, King Abdulaziz University, Jeddah 21589, Saudi Arabia; nalhakamy@kau.edu.sa (N.A.A.); smbali@kau.edu.sa (S.M.B.-E.); wsmalharbi@kau.edu.sa (W.S.A.); maalfaleh@kau.edu.sa (M.A.A.); omar.d.alhejaili@gmail.com (O.D.A.-h.); haldosari@kau.edu.sa (H.M.A.); rbakhaidar@kau.edu.sa (R.B.); 2 Advanced Drug Delivery Research Group, Faculty of Pharmacy, King Abdulaziz University, Jeddah 21589, Saudi Arabia; 3 Center of Excellence for Drug Research and Pharmaceutical Industries, King Abdulaziz University, Jeddah 21589, Saudi Arabia; 4 Mohamed Saeed Tamer Chair for Pharmaceutical Industries, King Abdulaziz University, Jeddah 21589, Saudi Arabia; 5 Department of Pharmaceutics and Industrial Pharmacy, Faculty of Pharmacy, Cairo University, Cairo 11562, Egypt; 6 Vaccines and Immunotherapy Unit, King Fahd Medical Research Center, King Abdulaziz University, Jeddah 21589, Saudi Arabia; 7 Department of Pharmacology and Toxicology, Faculty of Pharmacy, King Abdulaziz University, Jeddah 21589, Saudi Arabia; beid@kau.edu.sa; 8 Department of Biomedical and Biotechnological Sciences, University of Catania, 95123 Catania, Italy; fdrago@unict.it; 9 Department of Drug and Health Sciences, University of Catania, 95125 Catania, Italy; 10 Oasi Research Institute—IRCCS, 94018 Troina, Italy

**Keywords:** icariin, melittin, bilosomes, pancreatic cancer, apoptosis

## Abstract

Pancreatic cancer currently represents a severe issue for the entire world. Therefore, much effort has been made to develop an effective treatment against it. Emerging evidence has shown that icariin, a flavonoid glycoside, is an effective anti-pancreatic cancer drug. Melittin, as a natural active biomolecule, has also shown to possess anticancer activities. In the present study, with the aim to increase its effectiveness against cancerous cells, icariin-loaded bilosome-melittin (ICA-BM) was developed. For the selection of an optimized ICA-BM, an experimental design was implemented, which provided an optimized formulation with a particle size equal to 158.4 nm. After estimation of the release pattern, the anti-pancreatic cancer efficacy of this new formulation was evaluated. The MTT assay was employed for the determination of half maximal inhibitory concentration (IC_50_), providing smaller IC_50_ for ICA-BM (2.79 ± 0.2 µM) compared to blank-BM and ICA-Raw (free drug) against PNAC1, a human pancreatic cancer cell line isolated from a pancreatic carcinoma of ductal cell origin. Additionally, cell cycle analysis for ICA-BM demonstrated cell arrest at the S-phase and pre-G1 phase, which indicated a pro-apoptotic behavior of the new developed formulation. The pro-apoptotic and anti-proliferative activity of the optimized ICA-BM against PNAC1 cells was also demonstrated through annexin V staining as well as estimation of caspase-3 and p53 protein levels. It can be concluded that the optimized ICA-BM formulation significantly improved the efficacy of icariin against cancerous pancreatic cells.

## 1. Introduction

Worldwide, pancreatic cancer is currently reported as the seventh highest ranked cancer in terms of deaths, and it is more prominent in developing nations [1]. According to a large group of studies including global data, 432,242 deaths were due to pancreatic cancer in the year 2018 only [2,3], and this kind of cancer is expected to be the second main cause of cancer-related deaths by 2030 [4]. One of the reasons behind such statistics is the late-stage diagnosis; in fact, pancreatic cancer is a malignant digestive tract tumor, which is very difficult to diagnose in the early stages. Other reasons are the hidden clinical presentation, rapid progression, and poor treatment approaches [5].

As recently reported, icariin (ICA) possesses a prominent anticancer activity that has been related to the modulation of multiple pathways such as the inhibition of phosphatidylinositol 3-kinase (PI3K)/AKT and signal transducer and activator of transcription 3 (STAT3 pathways), the enhancement of p53 activity, and the induction of cell cycle arrest in S-phase [6,7,8,9]. Additionally, studies have shown the ability of ICA to remarkably inhibit the numbers of myeloid-derived suppressive cells in the spleen, which, in turn, leads to beneficial effects for cancer treatment [10]. Based on the above, ICA represents an effective asset against pancreatic cancer. Despite its promising anticancer activity, ICA suffers from the shortcomings of poor bioavailability due to poor solubility and permeability [11]. In this regard, nanocarriers can be of great help, improving the bioavailability of ICA by increasing the solubility and permeability across the biological membranes [12], which could enhance anticancer activity against pancreatic cancer.

In the present study, a novel nanocarrier represented by bilosomes, a bile salts-based niosomal system, was selected. Bilosomes are highly deformable and more flexible compared to previously reported nanocarriers such as liposomes and niosomes [13,14]. As natural detergents are part of this nanocarrier, bile salts are able to increase the permeability of drug molecules across biological barriers, also improving drug bioavailability at desired sites. Furthermore, the presence of bile salts in the lipid bilayer of a nanocarrier system protects the entrapped drug from the various gastrointestinal fluids [15]. Based on their multiple activities, bilosomes have been used as a drug delivery system in numerous studies [16,17,18].

Melittin (MEL) represents a major component of bee venom and has a unique three-dimensional structure due to the specific sequence of amino acids [19]. MEL has shown the ability to induce apoptosis in human leukaemia cells by interrupting the cell membrane with the release of tumor antigens. MEL also reduced the activation of epidermal growth factor receptor (EGFR) in her2-enriched and triple-negative breast cancer [20,21]. Therefore, in this study MEL was selected as a component with the potential to strengthen the anticancer activity of ICA against pancreatic cancer cells.

The aim of the present study was to investigate the cytotoxic and pro-apoptotic efficacy of the newly developed ICA-loaded bilosomes-MEL (ICA-BM) formulation in PNAC1, a human cancerous cell line often used to study the therapeutic potential of candidate molecules against pancreatic cancer [22,23,24]. A Box-Behnken experimental design was employed to obtain the optimized ICA-BM formula characterized by minimum vesicle size. After an in vitro release study, the optimized ICA-BM was examined in PNAC1 cells for the determination of half maximal inhibitory concentration (IC_50_) values, cell cycle, apoptosis and necrosis analysis, caspase-3 and p53 levels, and nitric oxide (NO) production.

## 2. Results

### 2.1. Preparation and Optimization of ICA-BM Using an Experimental Design

The different ICA-BM formulations were fabricated with the help of different selected excipients such as cholesterol, Span 20, bile salt, and MEL, all treated as independent variables for the Box-Behnken-based experimental design. After applying independent variables, the software suggested 15 different formulations with different ratios of excipients. Then all formulations were evaluated for particle size in order to select an optimized ICA-BM. Collected data were depicted in Table 1.

Table 2 depicts the statistical analysis of variance of response data, i.e., particle size.

The obtained *p*-value stated that all of the selected independent variables for the preparation of ICA-BM were statistically significant in impacting the particle size. The independent factor X1 showed a prominent impact on particle size. Additionally, independent factors X2 and X3 significantly influenced the particle size of ICA-BM. The R^2^ value was 99.6195, the adjusted R^2^ value was 98.9345, respectively, whereas the mean absolute error was 3.6444.

The software suggested a polynomial equation (Equation (1)) for particle size:

Size = 157.77 − 75.75 × X1 + 266.0 × X2 − 21.25 × X3 + 22.58 × X1^2^ + 0.0 × X1 × X2 + 13.5 × X1 × X3 − 250.67 × X2^2^ − 2.00 × X2 × X3 + 0.40 × X3^2^ (1)

**Figure 1:**
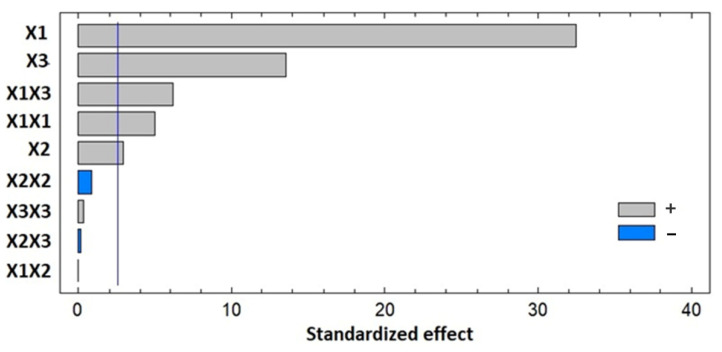
Preto chart for particle size of ICA-BM formulations, where X1, X2, and X3 represent the concentration of cholesterol:Span 20 ratio, bile salt, and MEL.

The effect of various independent variables on dependent variables (particle size) can be identified through the regression coefficient in the obtained polynomial equation. The positive sign for the regression coefficient for all of the selected factors indicates the positive effects of the factor on particle size and vice versa [25]. A regression coefficient value of +266.0 for bile salt indicates its significant contribution to increasing the particle size of the formulation. The Preto chart reported in Figure 1 shows the significant positive effects of independent factors and interconnected effects of cholesterol:Span 20, bile salt, and MEL on particle size of ICA-BM.

**Figure 2:**
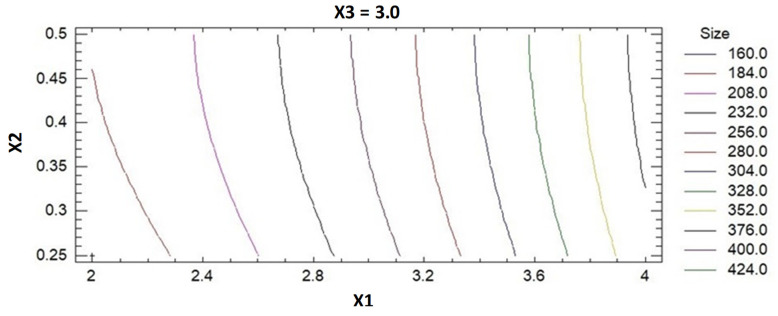
Contours of an estimated response surface for the particle size.

In Figure 2 are presented the contours of the analyzed response surface, which strengthen the results of the Preto chart.

### 2.2. Prediction of the Optimized ICA-BM Formulation

For the prediction of optimal ICA-BM formulation, a desirability function was applied based on the particle size predetermined goal (size minimization). The optimized formula was cholesterol:Span 20 molar ratio of 2.0 with 0.25 mM bile salt concentration and 1.14% *w*/*w* MEL concentration. The prepared optimized ICA-BM formulation was evaluated for actual particle size showing a mean value (*n* = 3) of 158.4 ± 0.93 nm that was in good agreement with the predicted value of 153.92 nm. The optimized formula was then subjected to the in vitro release and subsequent experiments with PANC1 cells.

### 2.3. In Vitro Drug Release Study

**Figure 3:**
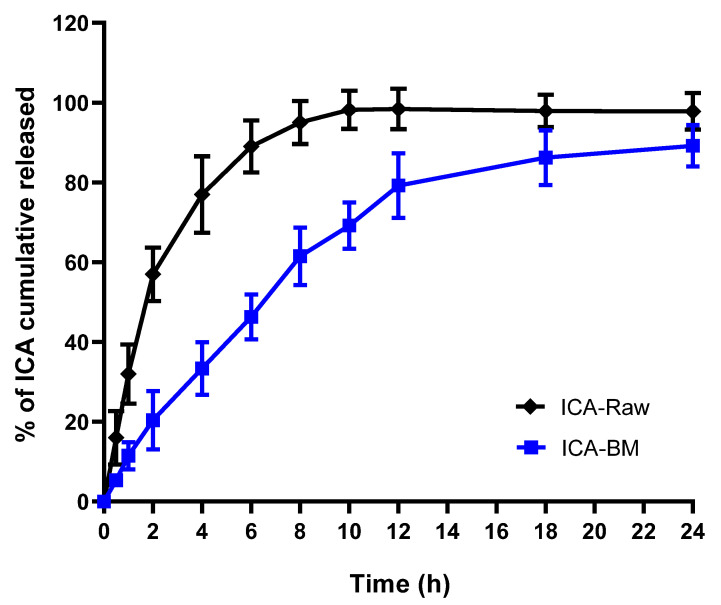
In vitro release profile of optimized ICA-BM.

The in vitro drug release of ICA from ICA-BM was carried out in phosphate-buffered saline (PBS) at pH 6.8. The release of ICA was estimated for 24 h, which indicated a sustained release pattern after the initial burst release (20.4 ± 7.3% at 2 h) (Figure 3).

89.2 ± 5.2% ICA was released from the ICA-BM after 24 h, indicating a sustained release mechanism.

### 2.4. Optimized ICA-BM Treatment Shows the Lowest IC_50_ Value

**Figure 4:**
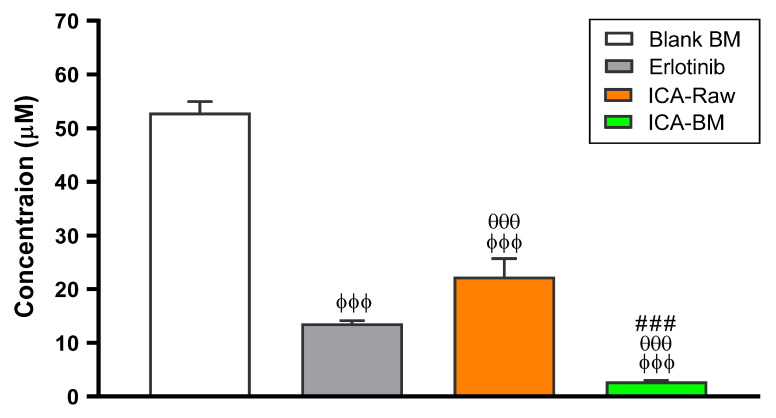
IC_50_ of the Blank BM, erlotinib, ICA-Raw, and ICA-BM in the PANC1 cells. Data are the mean of 4 independent experiments ± standard deviation (SD). ^ϕϕϕ^ Significantly different vs. Blank BM (*p* < 0.001); ^θθθ^ Significantly different vs. Erlotinib (*p* < 0.001); ^###^ Significantly different vs. ICA-Raw (*p* < 0.001).

Figure 4 reports the IC_50_ values obtained carrying out the MTT assay for PANC1 cells left untreated or treated with Blank BM, ICA-Raw, or ICA-BM for 24 h. The results show that the lowest IC_50_ value, indicating the highest toxic potential, was observed in cells treated for 24 h with ICA-BM (IC_50_ = 2.79 ± 0.2 µM; *p* < 0.001 vs. all).

Of note, the IC_50_ obtained for ICA-BM was even lower than erlotinib (13.62 ± 0.5), representing the positive control. The IC_50_ values obtained for blank-BM and ICA-Raw were 52.87 ± 2.1 and 22.3 ± 3.4, respectively.

### 2.5. ICA-BM Possesses an Enhanced Ability to Inhibit the Proliferation of PANC1 Cells

**Figure 5:**
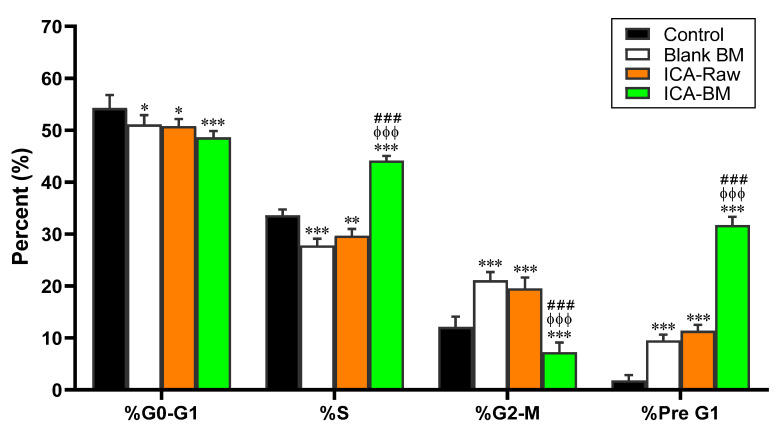
Effect of Blank BM, ICA-Raw, and ICA-BM on PANC1 cell cycle phases. Data are the mean of 4 independent experiments ± SD. * Significantly different vs. Control (*p* < 0.05); ** Significantly different vs. Control (*p* < 0.01); *** Significantly different vs. Control (*p* < 0.001); ^ϕϕϕ^ Significantly different vs. Blank BM (*p* < 0.001); ^###^ Significantly different vs. ICA-Raw (*p* < 0.001).

Figure 5 reports the effects of the different experimental conditions (Blank BM, ICA-raw, and ICA-BM) on PANC1 cell cycle phases.

As indicated by the percentage values measured in untreated cells with regard to G0/G1, S, G2-M, and Pre-G1 phases equal to 58.2 ± 2.5%, 33.6 ± 1.1%, 12.1 ± 2.0% and 1.8 ± 1.0%, respectively, PANC1 were characterized by quick proliferative properties in the absence of treatment. Blank BM and ICA-Raw treatments had a very similar effect on the different cell cycle phases of PANC1; in fact, both treatments were able to significantly decrease the percent of cells in the S phase (27.8 ± 1.3% for Blank BM, *p* < 0.001; 29.7 ± 1.3% for ICA-Raw, *p* < 0.01) paralleled by an increase of the number of PANC1 cells in G2-M (21.1 ± 1.6% for Blank BM, *p* < 0.001; 19.5 ± 2.1% for ICA-Raw, *p* < 0.001) and Pre-G1 (9.5 ± 1.1% for Blank BM, *p* < 0.001; 11.4 ± 1.1% for ICA-Raw, *p* < 0.001) phases compared to untreated cells. It is worth noting that the treatment of PANC1 with the optimized ICA-BM formulation was able to significantly inhibit the proliferation of these cells compared to all the other treatments, with significant and very relevant changes measured for the pre-G1 apoptotic phase (31.7 ± 1.6%), a percentage value three times higher than that observed for Blank BM (9.5 ± 1.1%) and ICA-Raw (11.4 ± 1.1%).

### 2.6. Optimized ICA-BM Treatment Increases Apoptotic and Necrotic Cell Populations

**Figure 6:**
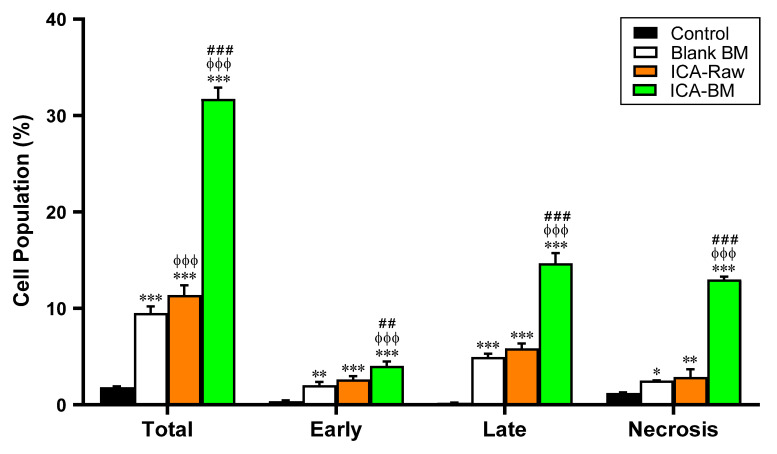
Effect of Blank BM, ICA-Raw, and ICA-BM on the percentage of apoptotic or necrotic PANC1 cells. Data are the mean of 4 independent experiments ± SD. * Significantly different vs. Control (*p* < 0.05); ** Significantly different vs. Control (*p* < 0.01); *** Significantly different vs. Control (*p* < 0.001); ^ϕϕϕ^ Significantly different vs. Blank BM (*p* < 0.001); ^##^ Significantly different vs. ICA-Raw (*p* < 0.01); ^###^ Significantly different vs. ICA-Raw (*p* < 0.001).

With the aim to better understand whether the potentiated anti-proliferative effect of ICA-BM treatment was also combined with pro-apoptotic and pro-necrotic activities, the effect of the different experimental conditions (Blank BM, ICA-raw, and ICA-BM) on the percentage of apoptotic or necrotic PANC1 cells was investigated. As observed for the cell cycle phase analysis, Blank BM and ICA-Raw treatments had a very similar effect on PANC1, significantly increasing the number of cells undergoing apoptosis (both phases) and necrosis (Figure 6).

As expected, based on our previous results, the treatment of PANC1 cells with ICA-BM significantly increased the percentage of cell population in early (4.0 ± 0.4%) and late (14.7 ± 1.1%) stages of apoptosis, in necrosis (13.0 ± 0.3%) as well as in apoptosis + necrosis (indicated as total) (31.7 ± 1.2%) compared to all the other experimental conditions (*p* < 0.05), demonstrating an enhancement of the pro-apoptotic and pro-necrotic activity of the optimized formula.

### 2.7. ICA-BM Treatment Strongly Increases Caspase-3 Activity in PANC1 Cells

**Figure 7:**
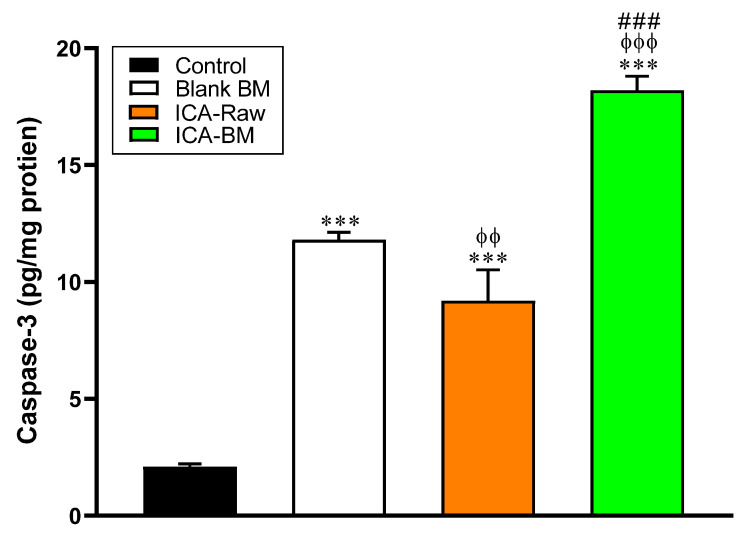
Effect of Blank BM, ICA-Raw, and ICA-BM treatments on caspase-3 enzyme content in PANC1 cells. Data are the mean of 4 independent experiments ± SD. *** Significantly different vs. Control (*p* < 0.001); ^ϕϕ^ Significantly different vs. Blank BM (*p* < 0.01); ^ϕϕϕ^ Significantly different vs. Blank BM (*p* < 0.001); ^###^ Significantly different vs. ICA-Raw (*p* < 0.001).

As clearly depicted in Figure 7, all the treatments significantly increased caspase-3 protein levels compared to control PANC1 cells (2.1 ± 0.1 pg/mg protein) (*p* < 0.001 for all treatments). As expected based on the results described in Figure 4, Figure 5 and Figure 6, the highest caspase-3 protein content was observed in PANC1 cells treatment with ICA-BM (18.2 ± 0.6 pg/mg protein) (*p* < 0.001 vs. all the other experimental conditions) that was about two and nine times higher than that of ICA-Raw-treated and control cells, respectively.

An intermediate level of caspase-3 protein content was found in cells treated with Blank BM (11.8 ± 0.3 pg/mg protein), being significantly higher than that of ICA-Raw (*p* < 0.01) and significantly lower than that of ICA-BM (*p* < 0.001).

### 2.8. The Levels of p53 Protein in PANC1 Cells Are Enhanced as a Consequence of ICA-BM Treatment

**Figure 8:**
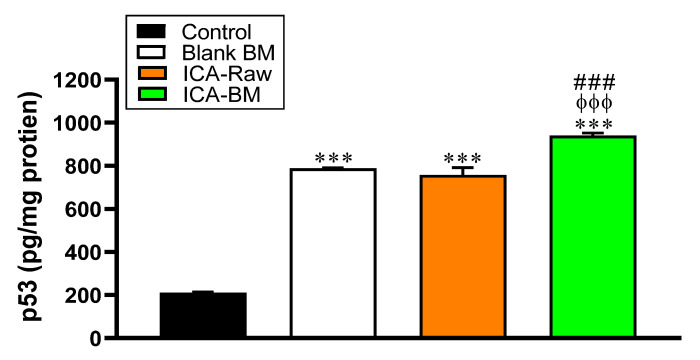
Effect of Blank BM, ICA-Raw, and ICA-BM treatments on p53 content in PANC1 cells. Data are the mean of 4 independent experiments ± SD. *** Significantly different vs. Control (*p* < 0.001); ^ϕϕϕ^ Significantly different vs. Blank BM (*p* < 0.001); ^###^ Significantly different vs. ICA-Raw (*p* < 0.001).

In this study, the effect of Blank BM, ICA-Raw, and ICA-BM treatments on p53 contents in PANC1 cells was also evaluated. In line with what observed in the case of caspase-3, all the treatments significantly increased p53 protein levels compared to control PANC1 cells (211 ± 3.3 pg/mg protein) (*p* < 0.001 for all treatments) (Figure 8).

Once again, the strongest inductive effect was observed in the case of ICA-BM optimized formulation, giving p53 protein content values (940 ± 12.0 pg/mg protein) significantly higher (*p* < 0.001) than that observed for Blank BM (788 ± 2.3 pg/mg protein) or ICA-Raw (757 ± 34.0 pg/mg protein).

### 2.9. NO Production Is Slightly Increased in PANC1 Cells Following ICA-BM Treatment

In order to investigate whether the observed anti-proliferative and pro-apoptotic/necrotic activities of the ICA-BM optimized formulation was also related to an increase of NO levels, the impact of the different treatments (Blank BM, ICA-Raw, and ICA-BM) on the production of NO by PANC1 cells was assessed. Differently from the previous set of experiments, no significant changes were observed in NO production between control cells and cells treated with Blank BM (+9%) or ICA-Raw (+3%). A higher (+21%), even though not significant (*p* > 0.05), change in NO production was observed for PANC1 cells treated with ICA-BM compared to control cells.

## 3. Discussion

ICA is a prenylated flavonol glycoside derived from the medical plant *Herba Epimedii* that has shown numerous pharmacological activities, including a potent antitumor activity in different types of cancer, including pancreatic cancer [26,27]. MEL is a natural active biomolecule that has also shown an anticancer activity against pancreatic cancer, inhibiting its proliferation and migration [28,29]. With this in mind, the aim of the present study was to developed a new ICA-loaded bilosomes-MEL (ICA-BM) formulation and investigate its cytotoxic and pro-apoptotic efficacy in PNAC1, a human cancerous cell line already used to test the therapeutic potential of candidate molecules against pancreatic cancer [22,23,24].

The optimized formula for the preparation of ICA-BM was obtained by employing the Box-Behnken-based experimental design coupled to the use of Statgraphics software [25]. In this design, the predicted and the adjusted R^2^ were less than the allowable limit 0.2, which certified the validity of the model of design. The Preto chart (Figure 1) demonstrated the positive effects of independent variables over the particle size of the newly formulated ICA-BM. As evidenced by the negative sign of X1, the particle size decreases significantly with increasing cholesterol:Span 20 ratio, i.e., higher proportion of cholesterol resulted in higher vesicular size. This could be attributed to the packing of cholesterol molecules between the surfactant alkyl chain, resulting in the higher size of the formed vesicles [30,31]. Additionally, it can be assumed that the increase in size with the increase in the concentration of MEL and bile salts could be attributed to the opposite charge attractions between these factors, with the possibility of integration within the geometrical structure of the formed vesicles. It has been previously reported that the increase in bile salts in the lipid vesicles increases the entrapment of the lipophilic drugs (as ICA) within the hydrophobic bilayer part of the lipid vesicles [32,33]. The optimized formula carried cholesterol:Span 20 at a molar ratio of 2.0 with 0.25 mM bile salt concentration and 1.14% *w*/*w* MEL concentration, which provided a particle size of 158.4 nm, in a very good agreement with the predicted value (153.92 nm). The data obtained by performing in vitro release (Figure 3) demonstrated a sustained release pattern; in particular, the initial burst release could be associated to the desorption of ICA from the bilosomes surface during the first 2 h, while the sustained release is ascribable to the high affinity of drug molecules with bilosomes [13,34].

The second part of the present study was dedicated to the investigation of the anticancer activity of the newly developed ICA-BM formulation. Our findings show that ICA-BM has an excellent and potentiated cytotoxicity against cancerous pancreatic cells compared to the free drug (ICA-Raw) (Figure 4, Figure 5, Figure 6, Figure 7 and Figure 8). The first evidence of a relevant improvement in the anticancer activity of ICA-BM is clearly demonstrated in Figure 4, in which the IC_50_ of this optimized formulation, a parameter often used to compare the antiproliferative activity and the toxic potential of different antineoplastic drugs [35], is equal to 2.79 ± 0.2 µM, a value ten times lower compared to that of ICA-Raw (22.3 ± 3.4). Most probably the observed enhanced cytotoxic effects observed when employing the optimized formula are coming from the ability of BM to enhance the poor solubility of ICA, ensuring a higher intracellular availability [12]; in fact, despite its promising anticancer activity, ICA is characterized by poor bioavailability due to low solubility and permeability [11]. The relevance of this result is also connected to the fact that cancer patients often encounter severe adverse effects attributable to the necessity of administering high doses of the drug [36], so the development of new formulations able to maximize the clinical efficacy, then reducing the doses, is of great importance. ICA-BM also demonstrated an enhanced ability to inhibit the proliferation of PANC1 cells (Figure 5). In particular, the optimized ICA-BM significantly enhanced the percentage of cells in pre-G1 phase (31.7 ± 1.6%), representing an indicator of apoptosis [37] compared to all the other experimental conditions, including ICA-Raw (*p* < 0.001), paralleled by increased S-phase arrest. These results are also in agreement with previous findings showing that the optimized formulation of ICA into phytosomes enhanced the cytotoxicity and apoptosis-inducing activities of the drug in ovarian cancer cells [38]. It is worth emphasizing that despite a reduced antineoplastic activity compared to the optimized formula, the free drug (ICA-Raw) significantly increased the number of cells in pre-G1 phase compared to untreated cells (*p* < 0.001). Our results are in accordance to previous studies showing the ability of ICA to remarkably decrease the cells in the S-phase, in particular in liver hepatocellular carcinoma cells (HepG2) [39] and airway smooth muscle cells (ASMCs) [40]. Of note, ICA has also shown an opposite effect; therefore the induction of cell cycle arrest in S-phase in lung cancer cells (A549) [6] and medulloblastoma (D341) [41], demonstrating a different behavior based on the type of cancer considered. With regard to cell cycle modulation, two additional factors that could affect the antineoplastic activity of ICA are represented by the concentration used and the duration of the treatment; in fact, we employed a very low concentration (sub-toxic, based on IC_10_) of ICA-Raw as well as a shorter treatment time (24 h) compared with the conventional experimental conditions used (ICA at µM concentration and treatment time ≥ 48 h).

As indicated by our results reported in Figure 6, the anti-proliferative activity demonstrated by the optimized formula was paralleled by a strong enhancement of the pro-apoptotic/necrotic potential, as also strengthened by the increased caspase-3 (18.2 ± 0.6 pg/mg protein, Figure 7) and p53 (940 ± 12.0 pg/mg protein, Figure 8) content measured after the treatment of PNAC1 cells with ICA-BM. As observed in different research studies, antiproliferative activity and induction of apoptosis are two mechanisms correlated and often simultaneously activated by molecules showing anticancer activity [42,43,44]. Caspase-3 is considered a “gold standard” marker of apoptosis because, during this process, it regulates the cellular protein destruction as well as the fragmentation of DNA [45]. Interestingly, Blank BM exhibited an enhanced ability to induce caspase-3 compared to ICA-Raw, and it might be due to the already known pro-apoptotic effect of MEL part of the carrier [20,21]. With regard to p53 protein, our results are in agreement with previous studies showing how ICA exerts its anticancer activity through the amplification of p53 activity [8]. The reason behind the significant and improved anticancer activity of the optimized formulation could also be ascribable to the enhanced bioavailability of ICA made possible by the ultra-flexible behavior of bilosomes [13,38]. As previously described, no significant changes were observed in NO production among our experimental conditions, even though a trend (+21%) was observed for PANC1 cells treated with ICA-BM compared to control cells. Increasing the concentration of the stimulants and/or prolonging the stimulation time could lead to a more significant increase in NO production, especially in the case of the optimized formula. Additionally, in our experiments we used epithelial cells (PANC1) that are less responsive than immune cells where the positive modulation of the inducible form of NO synthase (iNOS) is related to a very high production of NO [46,47].

Overall, our results clearly show the augmented cytotoxic activity exerted by ICA drug when encapsulated into a bilosomes-MEL carrier on the well-characterized human PNAC1 cells, setting the stage for the investigation of optimized ICA-BM formula in vivo.

## 4. Materials and Methods

### 4.1. Materials

Cholesterol, ICA, MEL, sodium deoxycholate, chloroform, methanol, and acetonitrile were obtained from Sigma-Aldrich Inc. (St. Louis, MO, USA). Pancreatic cells (PANC1) were obtained from National Centre for Cell Science (NCCS) (Pune, India). All chemicals were supplied by Thermo Fisher Scientific Inc. (Pittsburgh, PA, USA) unless specified otherwise. All the reagents and chemicals used in the present study were of analytical grade.

### 4.2. Experimental Design

The Box-Behnken experimental design was utilized to formulate the ICA-BM by employing Statgraphics plus software version 4 (Statgraphics Centurion XV version 15.2.05, Statgraphics Technologies, Inc., Warrenton, VA, USA) [25]. Three different variables were taken into consideration: cholesterol:Span 20 molar ratio (X1), bile salt molar concentration (X2, mM), and MEL concentration (X3, %*w/w*), and their effects were analyzed on the response and particle size (Y). After taking these independent variables into their constraints via software (Table 3), 15 experimental trials with three center points were obtained (Table 1).

### 4.3. Formulation of ICA-BM

A modified thin-film hydration method was employed to formulate ICA-BM, as previously described [15]. Briefly, ICA (20 mg), cholesterol, and Span 20 (amount indicated in the experiment’s design) were dissolved in 10 mL of chloroform. Then, at a temperature of 65 °C and under reduced pressure, the organic solvent was evaporated from the solution by utilizing a rotary evaporator until dry and thin film was obtained. Next, the defined quantity (according to the design) of MEL was dispersed in the double-distilled water (5 mL) according to the data obtained from the experimental design. MEL solution was added to the aqueous solution of bile salts (sodium deoxycholate as surfactant, according to the design), followed by hydration. The aqueous mixture was hydrated under rotation (1 h) to obtain a dried film, ultra-sonicated (Sonics & Materials Inc., Newtown, CT, USA), and kept at 4 °C until use.

### 4.4. Particle Size Evaluation of ICA-BM Formulations

The particle size of fabricated ICA-BM nanoformulations, required for the experimental design, was evaluated using a Zetasizer Nano ZSP (Malvern Panalytical Ltd. Malvern, UK). The samples of different fabricated ICA-BM dispersions were prepared by dilution with appropriate volume (1.5 mL) of double-distilled water and then measured [48].

### 4.5. Prediction of the Optimized ICA-BM Formulation

The evaluated ICA-BM fabrication parameters were optimized using a numerical standpoint, thereupon a desirability technique. The received data from 15 different prepared nanoformulations were assessed by design experiment software employing ANOVA, followed by the multiple response optimization. As a result, the ideal quantity for the selected three independent variables was established. This optimization method was established with the aim of formulating the ICA-BM with minimum particle size. Further, the predicted optimized ICA-BM was fabricated and evaluated for release and efficacy.

### 4.6. ICA In Vitro Release from the Optimized ICA-BM

The rate of release for ICA from BM was analyzed as per the previously reported methods [15,49]. For this purpose, 0.1 M PBS at pH 7.4 containing Tween 80 (0.1%) to maintain the sink condition was used. A volume of ICA-BM containing 5 mg of ICA was loaded in a previously activated dialysis bag with 12,000 Da molecular weight cut-off. The temperature of the experimental area was maintained at 37 °C in a water bath shaker at 100 rpm. Then, samples were collected at the different prescribed time period (0.5, 1, 2, 4, 6, 8, 10, 12, 18, and 24 h) and analyzed in triplicate by using a HPLC method [38,50].

### 4.7. Determination of IC_50_ by MTT Assay

PANC1 cells were cultured as previously described [51]. The IC_50_ values of human pancreatic cancer cells treated with Blank BM, erlotinib (used as a positive control [52]), pure ICA (ICA-Raw), or ICA-BM for 24 h, were measured by the MTT [3-(4,5-dimethylthiazol-2-yl)-2,5-diphenyltetrazolium bromide] assay as previously described [53]. A 96-well culture plate was used in which PANC1 cells (1 × 10^5^ cells) were seeded and incubated in a humified condition (5% CO_2_, 37 °C) to confirm the absolute attachment of the cells [54]. At the end of the 24 h treatment, the MTT protocol was applied and the absorbance at 569 nm was read by using a microplate reader (Spark^®^ multimode, Tecan Group Ltd., Seestrasse, Maennedorf, Switzerland). The IC_50_ for Blank BM, erlotinib, ICA-Raw, or ICA-BM was calculated based on the curves obtained measuring the variation of cell viability (%) as a function of increasing concentrations (0.39, 1.56, 6.25, 25, and 100 µM) of Blank BM, erlotinib, ICA-Raw, or ICA-BM.

### 4.8. Cell Cycle Analysis

For the cell cycle analysis, PANC1 cells were seeded in 6-well culture plates (3 × 10^5^ cells/well) and left untreated (control) or treated with Blank BM, ICA-Raw, or ICA-BM for 24 h. A sub-toxic concentration of the different treatments was selected based on the IC_10_ (the concentration giving a response of 10%). At the end of the treatment, cells were separated via centrifugation and fixed with 70% cold ethanol. Samples were centrifuged again, washed with PBS, and then stained by using the propidium iodide and RNase staining buffer mixture. Finally, every treated sample was analyzed through a FACS caliber flow cytometer (FACS Calibur, BD Bioscience, San Jose, CA, USA) [55].

### 4.9. Apoptotic Analysis by Annexin V Staining

PNAC1 cells were seeded in 6-well culture plates (3 × 10^5^ cells/well) and incubated under the four above-mentioned experimental conditions for 24 h. The Annexin V-FITC Apoptosis Kit (BD Bioscience) was used to study the effects of the different treatments and distinguish between early and late apoptosis as well as to differentiate apoptosis from necrosis. After completion of the incubation period, cells were centrifuged, washed by using PBS, and resuspended in 500 µL of 1× binding buffer. Next, staining of cells was obtained by adding 5 μL of both propidium iodide and Annexin V-FITC to each well and incubating for 5 min at room temperature in a dark environment followed by flow cytometry analysis. Lastly, samples were analyzed through flow cytometry, while the MultiCycle AV software (Phoenix Flow Systems, San Diego, CA, USA) was employed for the analysis of the obtained data [56].

### 4.10. Caspase-3 Enzyme Assay

Caspase-3 activity in PNAC1 cells cultured as in the case of cell cycle analysis and left untreated (control) or treated with Blank BM, ICA-Raw, or ICA-BM for 24 h was measured by using a commercial kit (USCN Life Science Inc., Wuhan, China) following manufacturer’s instructions. At the end of the treatment, cells were lysed by using a cell extraction buffer, and 100 µL sample from each treatment was used for assaying caspase-3 by reading the absorbance at 450 nm with a Spark^®^ multimode microplate reader (Tecan Group Ltd., Seestrasse, Maennedorf, Switzerland).

### 4.11. Quantification of p53 Protein Levels

In pancreatic cancer, the tumor protein p53 is mutated and accelerates the tumorigenesis in 75% of cases [57]. Therefore, p53 tumor protein was selected as a biomarker in this study. For this purpose, 96-well culture plates were used in which PANC1 cells (5 × 10^4^ cells/well) were seeded and incubated. Cells were left untreated (control) or treated with Blank BM, ICA-Raw, or ICA-BM for 24 h. After completion of the incubation period, p53 tumor protein in each type of sample was quantified by using an ELISA kit (Invitrogen^®^, Thermo Fisher Scientific, Waltham, MA, USA) following manufacturer’s instructions [25].

### 4.12. Evaluation of NO Level

The level of NO was indirectly estimated measuring the stable NO end-product, nitrite, by using the Griess assay. PANC1 cells were seeded in 96-well culture plates (1 × 10^4^ cells/well) and incubated in a humified condition (5% CO_2_, 37 °C). The day of the treatment, cells were left untreated (control) or treated with Blank BM, ICA-Raw, or ICA-BM for 24 h. At the end of the treatment, for extracellular nitrite quantification, 100 μL of supernatant were taken from each well and added to 100 μL of Griess reagent. After 15 min at room temperature in the dark, the absorbance was measured at 540 nm by using a microplate reader (BMG Labtech, Ortenberg, Germany).

### 4.13. Statistical Analysis

The obtained data were statistically assessed through the IBM Statistical Package for Social Science (SPSS) software (version 25) (SPSS Inc., Chicago, IL, USA). The mean value of the various outcomes was compared by employing ANOVA followed by Tukey’s *post hoc* test [58]. Only values of *p* < 0.05 were considered statistically significant [59].

## 5. Conclusions

In this study, optimized ICA-BM was obtained through implementing a Box-Behnken-based experimental design by using Statgraphics software, which provided a formulation with optimum particle size. The in vitro release study demonstrated a sustained release pattern for ICA from ICA-BM. The in vitro experiments carried out on the human pancreatic cancer cell line (PNAC1) clearly demonstrated that the loading of drug (ICA) into a bilosomes-MEL carrier (ICA-BM) significantly improved all the parameters related to the anticancer efficacy of the ICA towards cancer cells, including the decreased IC_50_, the enhanced anti-proliferative and pro-apoptotic/necrotic activities, as well as the augmented caspase-3 and p53 protein levels. All together these findings indicate that the optimized ICA-BM formulation might represent a novel path for the development of a specific and very effective treatment for pancreatic cancer.

## Figures and Tables

**Table 1:** The obtained particle size from various trials by Box-Behnken experimental design for the ICA-BM.

	Factors			Response
Run	X1	X2	X3	Size (nm)
1	2.0	0.375	5.0	198
2	3.0	0.25	1.0	208
3	3.0	0.375	3.0	265
4	3.0	0.375	3.0	264
5	4.0	0.5	3.0	387
6	4.0	0.25	3.0	365
7	4.0	0.375	5.0	453
8	2.0	0.5	3.0	187
9	3.0	0.5	1.0	223
10	2.0	0.375	1.0	164
11	3.0	0.5	5.0	301
12	3.0	0.375	3.0	243
13	4.0	0.375	1.0	311
14	3.0	0.25	5.0	288
15	2.0	0.25	3.0	165

X1: cholesterol:Span 20 molar ratio; X2: bile salt molar concentration; X3: MEL concentration.

**Table 2:** Statistical analysis of variance (ANOVA) of the responses (particle size).

Effect	Estimate	F-Ratio	*p*-Value
X1	200.5	1053.28	0.0000
X2	18.0	8.49	0.0333
X3	83.5	182.68	0.0000
X1X1	45.1667	24.67	0.0042
X1X2	0.0	0.00	1.0000
X1X3	54.0	38.20	0.0016
X2X2	−7.83333	0.74	0.4284
X2X3	−1.0	0.01	0.9133
X3X3	3.16667	0.12	0.7419
R^2^			99.6195
Adjusted R^2^ (adjusted for degree of freedom)			98.9345
Standard Error of estimated			8.73689
Mean absolute error			3.64444

**Table 3:** Factors involved in Box-Behnken design and their selected levels with the response and their constraints and goals.

Factor	Low	High	Units
**X1:** cholesterol:Span 20 molar ratio	2	4	
**X2:** bile salt molar concentration	0.25	0.5	(mM)
**X3:** MEL concentration	1.0	5.0	(%*w/w*)
**Response**	Low	High	Goal
**Size** (nm)	164	453	minimize

## Data Availability

The data presented in this study are available in article.
